# Evaluation of Physical Activity and Lifestyle Interventions Focused on School Children with Obesity Using Accelerometry: A Systematic Review and Meta-Analysis

**DOI:** 10.3390/ijerph17176031

**Published:** 2020-08-19

**Authors:** Jose Manuel Jurado-Castro, Mercedes Gil-Campos, Hugo Gonzalez-Gonzalez, Francisco Jesus Llorente-Cantarero

**Affiliations:** 1 Metabolism and Investigation Unit, Maimonides Biomedical Research Institute of Cordoba (IMIBIC), Reina Sofia University Hospital, University of Cordoba, 14004 Córdoba, Spain; juradox@gmail.com (J.M.J.-C.); llorentefj@yahoo.es (F.J.L.-C.); 2 CIBEROBN, (Physiopathology of Obesity and Nutrition), Institute of Health Carlos III (ISCIII), 28029 Madrid, Spain; 3 Unit of Metabolism and Pediatric Research, Reina Sofia University Hospital, 14004 Córdoba, Spain; 4 Department of Education, University of Cordoba, 14071 Córdoba, Spain; hugo.gonzalez@uco.es; 5 Department of Specific Didactics, Faculty of Education, University of Cordoba, 14071 Córdoba, Spain

**Keywords:** active life, accelerometry, child, exercise, schools

## Abstract

Despite the existence of global recommendations for physical activity and lifestyle to avoid childhood obesity, there are no specific recommendations for school-age children. The aim of this meta-analysis was to measure the effects of current interventions with a physical activity component on body mass index (BMI) Z-score and on the moderate and vigorous physical activity (MVPA) time, measured by accelerometry, and focused on children with obesity. Randomized controlled trial studies (RCTs) based on physical activity interventions focused on children with obesity (6 to 12 years old) from January 1991 to August 2018 were included. The post-intervention mean and standard deviation of the BMI Z-score and MVPA engaged time were extracted to calculate the results using random effects models. Of a total of 229 studies considered potentially eligible, only 10 RCTs met the inclusion criteria. There were improvements in the BMI Z-score for physical activity intervention groups, compared with non-intervention children in addition to a significant increase in time engaged in MVPA. In conclusion, interventions with a physical activity component in school-children with obesity seem to be effective at reducing BMI and producing an increase in time spent engaged in physical activity. Therefore, interventions based on physical activity should be considered one of the main strategies in treating childhood obesity.

## 1. Introduction

Epidemiological studies continue to show an alarmingly increasing prevalence of childhood obesity in developed countries, despite the different strategies being carried out by governments [1]. It is well known that obesity increases the risk of cardiovascular diseases, and it is associated with physical and mental health problems in both children and adults [2]. In this regard, traditional obesity treatment in children tries to promote a healthier lifestyle and good nutritional habits by increasing physical activity (PA) and modifying behavior, such as walking to school. However, there is no specific information about the effects these interventions have on children with obesity [3,4,5], for example, the length, intensity and frequency per week of the sessions, weekend sessions or exercise type. Some reviews [6,7,8,9,10,11] have considered individual studies which examined behavioral interventions regarding weight control, obesity prevention or treatment. These included PA, dietary patterns or a combination of both and concluded that there was only limited evidence about specific recommendations. Moreover, most of these exercise- or PA-based interventions are obtained through questionnaires and not measured objectively using accelerometry [9]. Currently, accelerometry is the best tool to measure the time spent in sedentary and PA behaviors. It can also estimate the intensity levels of different evaluated periods [12]. In addition, variables such as age, sex, pubertal stage or BMI of the selected subjects, as well as the different intervention types and results, lead to high heterogeneity [6]. A Cochrane review [13] concluded that behavioral lifestyle interventions, which often include a multidisciplinary component in treating childhood obesity, could be effective in obtaining a significant reduction in overweight. However, the intervention effects on PA were not considered.

At present, regular PA practice, starting during childhood, appears to help to maintain a healthy metabolic status and seems to be an effective tool in treating childhood obesity [14]. This is especially true during growth [15] where it has been found to be associated with lower morbidity and mortality rates in adulthood [16]. Particularly in children and adolescents affected by obesity, it can induce a positive adjustment in adiposity tissue, regardless of weight loss [17]. In fact, since 2010, the World Health Organization (WHO) has recommended at least 60 min of daily moderate to vigorous PA (MVPA) engagement for children and young people (5–17 years) to improve health [18]. However, the percentage of children achieving these recommendations still remains very low [19] despite multiple protocols and interventions being carried out. This may be partly explained by the absence of specific recommendations for children at the prepubertal stage [20,21].

Addressing the alarming reduction in PA practice amongst children [19] is the main priority of the present programs to tackle childhood obesity. Therefore, an objective method is necessary to examine the daily routine of children and to be able to promote a more active lifestyle at different life stages. A particularly important group to target is that of school-aged children [8,9,22]. Under this premise, studies have been carried out on the effectiveness of interventions with a main component of PA measured objectively [7,23]. However, these interventions achieved small or negligible increases in PA for children and adolescents, as well as limited success in reducing BMI or body fat, or were carried out mainly in adolescents. Furthermore, it seems that subgroup analysis where interventions are aimed exclusively at an overweight or obese population tended to be slightly more effective compared to those aimed at all children [23].

After these findings, and due to the fact that the current evidence includes adolescents in its results, which may be confounding factors for younger children, it remains unknown how the interventions directed at school-age children with obesity affect them specifically. Thus, the aim of the present study is to evaluate the effect of current interventions with a PA component on body mass index (BMI) and time spent engaged in MVPA via accelerometry measurement, among school-age children with obesity to establish better practices that promote adherence to a more active lifestyle.

## 2. Materials and Methods

A systematic review and meta-analysis of published studies was conducted using the Preferred Reporting Items for Systematic Reviews and Meta-Analysis (PRISMA) [24] (Table S1). The pre-defined review protocol was registered at the PROSPERO (International Prospective Register of Systematic Reviews), registration number CRD42020095179.

### 2.1. Criteria for Considering Studies for Inclusion in the Review

This systematic review included randomized controlled trial studies (RCTs) published between January 1991 and August 2018.

The inclusion criteria were established according to the PICO(S) outline: P (population): Samples of school age children (6 to 12 years old) and also subsamples including children with obesity; I (intervention) and C (comparison): PA interventions objectively measured using accelerometry taking into consideration a MVPA evaluation versus a control group; O (outcome): Reduction of the BMI Z-Score and time spent engaged in MVPA; (S) (study type): Randomized controlled trials studies.

Exclusion criteria were non-primary studies (for example, letters and reviews of narrative literature; duplicate publications); studies conducted on children not in the 6 to 12 age range; studies not including children with obesity and a PA component; studies in which the PA data were not accelerometry-quantified; and studies in which the MVPA or BMI data were not reported.

### 2.2. Protocol for Electronic Searching

The search of scientific literature was performed in electronic databases of published articles such as MEDLINE (PubMed), Cochrane Register of Controlled Trials (CENTRAL), and Web of Science. A complementary search was carried out to explore other secondary national and international databases. These included ScienceDirect (SCOPUS), PROQuest, BVS (Biblioteca Virtual en Salud), Annual Reviews, LILACS (Literatura Latino Americana y del Caribe en CC de la Salud), Dialnet, and Scielo. The search strings consisted of key words related to “physical activity”, “accelerometry”, “child”, and “pediatric obesity” which are presented in the Supplementary Material. All identified studies were then critically examined to identify potentially eligible papers.

### 2.3. Study Selection and Data Collection

Two independent reviewers conducted the searches and analyzed the studies (J.M.J.-C. and F.J.L.-C.). Articles found were coded using the reference manager RefWorks [25], and discrepancies regarding the interpretation of the extracted data were discussed by both investigators. Moreover, the articles were filtered using the inclusion criteria. The dropout rate was studied for each study (<20%).

The search was divided into two phases. During the first search, articles were selected according to their title and summary/abstract. Articles that did not meet the inclusion criteria were discarded. In the second phase, the entire article was read and analyzed. Information from the articles was extracted regarding the number of participants, the countries in which the research had been carried out, the type and duration of the interventions, the percentage of both genders, and whether the studies included only children with obesity. In addition, specific data with regard to the use of accelerometry in each intervention were evaluated.

### 2.4. Risk of Bias in Individual Studies

Evaluation of the risk of bias was carried out following the recommendations of the Cochrane Collaboration [26]. For each study, seven domains were scored as having a high, low or unclear risk of bias. These domains were: sequence generation, allocation concealment, blinding of participants and personnel, blinding of outcome assessment, incomplete outcome data, selective outcome report, and other considered issues.

### 2.5. Statistical Analysis

A random-effects model method was used to measure the effect of the included studies, as it was more appropriate than a fixed-effects model due to the sample heterogeneity of the analysis. To perform the meta-analysis, the effect of the interventions with a principal component of PA was examined on BMI Z-score reduction and MVPA increase, comparing intervention with control groups. Data were obtained via mean and standard deviation from post-intervention selected data.

Another analysis was carried out using a fixed-effects analysis with studies only including children with obesity, due to sample homogeneity.

Some studies developed a PA intervention combined with nutritional recommendations. Therefore, a subgroup analysis was carried out to measure the effect of these interventions when focused on BMI Z-score reduction.

Meta-analysis outcomes were presented in forest plots as mean differences (MD) and 95% confidence interval (CI); each arm of a multi-arm study was presented separately. Heterogeneity (I^2^) was also presented. Heterogeneity was calculated by measuring its scope by the I^2^ index. The Q statistics were estimated, following a chi-square distribution with degrees of freedom n − 1 (n = number of studies included in the analysis). The authors examined the value of P for this statistic, warning of the presence of heterogeneity when *P* < 0.05, which compromises the validity of the pooled estimates [27]. Therefore, possible outliers were examined and sensitivity analysis was performed to explore the effect of removing some studies with results classified as outliers. After adjusting the sensitivity analysis, studies considering candidates acting as effect modifiers were excluded [28].

The available numeric data were extracted by the authors in Review Manager (RevMan, computer program) version 5.3. [29]. A value of *P* < 0.05 indicated statistical significance in all analyses. Results are shown in mean values followed by another value with the symbol ‘±’ (representing the standard deviation).

## 3. Results

### 3.1. Studies Selected

**Figure 1:**
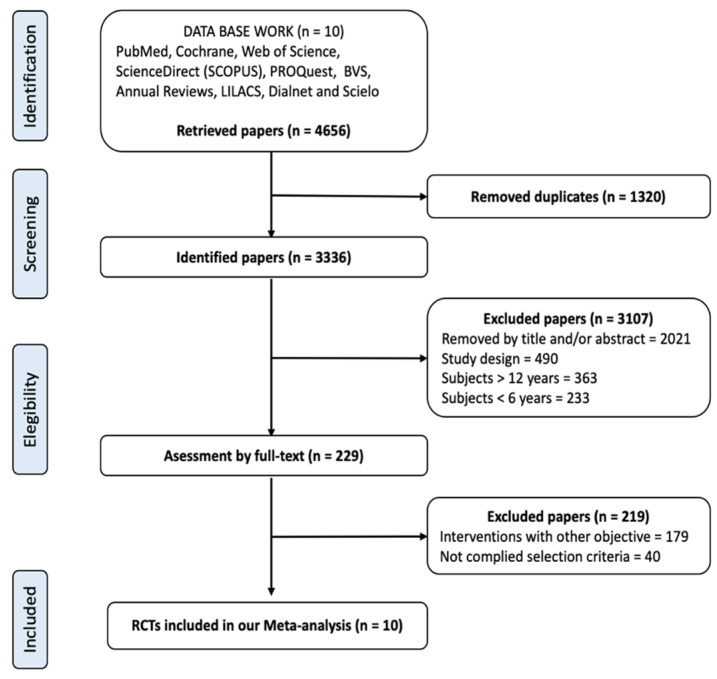
Flow diagram for the scientific paper selection from databases. RCTs, randomized controlled trials.

A flow chart diagram describes the selection of articles that were included in this meta-analysis (Figure 1). A total of 4656 papers were identified from the various included databases, following the review. A total of 1320 articles were deleted as duplicates, leaving a potential of 3336 papers to be selected for inclusion. A total of 3107 articles were eliminated for the following reasons: (1) the title or abstract was not associated with the aims; (2) they were not RCTs; or (3) the subjects did not meet the age criteria. Following this, 229 full text articles were considered to be potentially eligible according to the inclusion criteria. However, 179 were eliminated because the interventions did not include PA measures, and 40 were discarded because they did not include children with obesity in their samples. Thus, 10 articles were finally selected for the present meta-analysis, of which 4 studies included only children with obesity (Table 1).

### 3.2. Description of Selected Studies

The characteristics of the included RCTs are provided in Table 1. Four of the selected studies were carried out in the USA [30,31,32,33], two in New Zealand [34,35], one in Switzerland [36], others in Australia [37] and Malaysia [38], and one in Spain [39]. A total of 478 children (9 ± 1.57 years) participated in a PA intervention compared to 474 (9 ± 1.62 years) who were recruited to a control group without intervention. The dropout rate was studied for each intervention; a 16.3% average was obtained. The average intervention duration was 8.4 ± 6 months. Most of the PA interventions were accompanied by a lifestyle educational intervention, where the principal aim was to increase activity time and promote healthy behavioral habits [30,31,39]. Interventions with a nutritional component were based mainly on recommendations [32,35,37,38]. Alternatively, active video games (AVG) were incorporated [33,34]. Lifestyle education was included in six of these interventions.

To define obesity, different criteria were used in all of the included studies. Four studies [32,33,35,38] used the cut-off points of the Centers for Disease Control and Prevention of United States [40], three studies [34,37,39] went with the International Obesity Task Force [41], another study [36], applied Kromeyer-Hauschild et al. [42], and in other cases [30,31], nothing was referred to. The BMI Z-score ranged between 1.72 ± 0.52 and 2.92 ± 0.65 in the intervention groups, and between 1.55 ± 0.59 and 3.00 ± 0.49 in non-PA intervention groups.

Different protocols [43,44,45,46,47,48,49,50,51] in accelerometry measurements were described (Table 1). The ActiGraph was the most commonly used device to quantify PA, especially the GT3X + model. Wear time ranged between 5 [32,38] and 8 days [35,37,39]. The time spent engaged in MVPA ranged from 20.7 min [30] to 104.3 min [31] for the intervention groups. In contrast, engagement in the control groups was reported to range between 17.2 min [30] and 83.3 min [34] of MVPA.

### 3.3. Risk of Bias in Included Studies

Within the seven domains established to analyze the risk of bias, only three present noteworthy aspects. After comprehensively reading all of the selected articles, it remained unclear whether the blinding of participants, personnel, and outcome data had been correctly carried out for eight of the articles [30,31,32,33,34,36,38,39]. Furthermore, the inclusion of normal weight or overweight children as part of the intervention group resulted in a high risk of bias in six studies [30,33,34,35,37,39] (Figure S1).

### 3.4. Effects of the Interventions

To measure the effect of PA interventions, the BMI Z-score and time spent engaged in MVPA were analyzed. Of the 10 articles selected, two studies were excluded because they did not present BMI results as a Z-score [37,39]. Moreover, a further four studies were excluded from the MVPA analysis: Three showed the data as counts per min [36,37,38], and the other only showed the percentage of MVPA time [39].

#### 3.4.1. BMI Reduction

**Figure 2:**
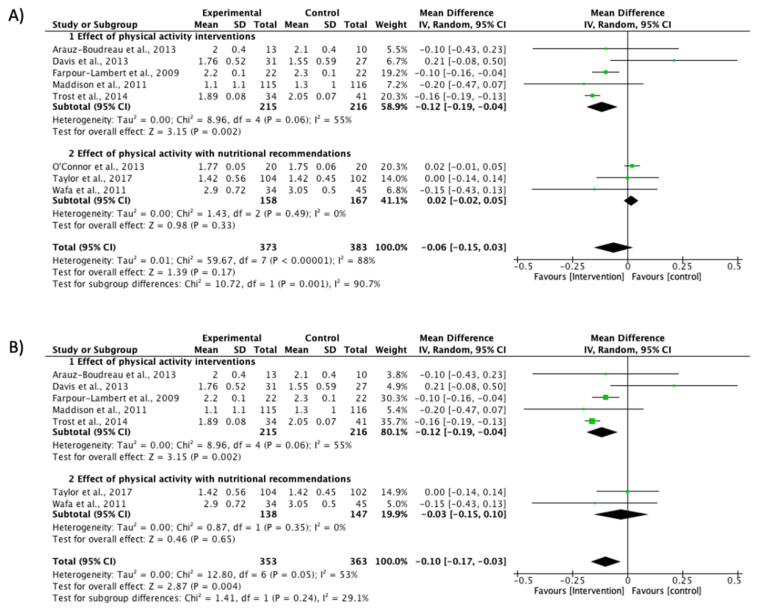
Effect interventions on BMI Z-score reduction. (**A**) BMI Z-score values forest plot. (**B**) Sensitivity analysis of BMI Z-score forest plot (O’Connor et al. study [32] not included). CI, confidence interval.

The BMI Z-score was measured in eight studies included in the meta-analysis (MD −0.06; CI −0.15 to 0.03; *P* = 0.17; I^2^ = 88%) (Figure 2A). After a sensitivity analysis, O’Connor’s study [32] was excluded to reduce heterogeneity to a moderate level (MD −0.10; CI −0.17 to −0.03; *P* = 0.004; I^2^ = 53%) (Figure 2B). The first section included studies with PA intervention as the principal component [30,31,33,34,36], and a significant effect was found in the reduction of this variable (MD −0.12; CI −0.19 to −0.04; *P* = 0.002; I^2^ = 55%). Trost et al. [33] presented the highest proportion in this meta-analysis (35.7%), followed by Farpour-Lambert et al. [36]: (30.3%) (Figure 2B). The second section included interventions with nutritional recommendations [35,38] without effects (MD −0.03; CI −0.15 to 0.10; *P* = 0.65; I^2^ = 0%).

**Figure 3:**
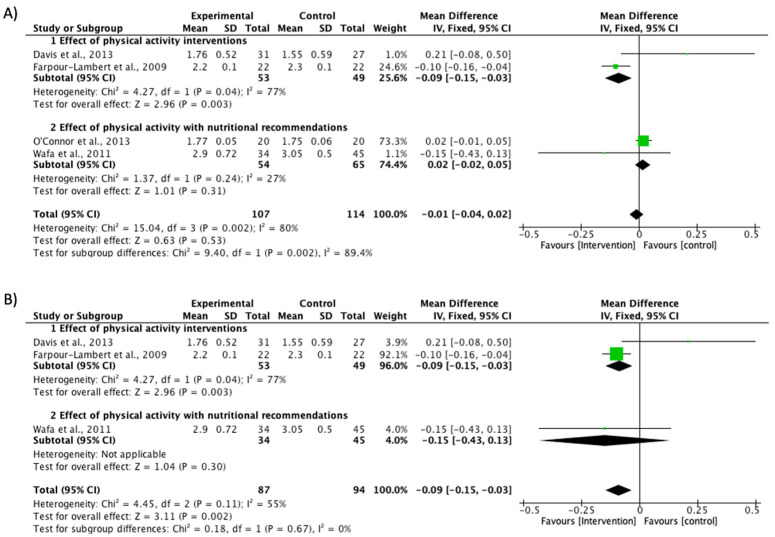
Effect interventions on BMI Z-score reduction in studies only including children with obesity. (**A**) BMI Z-score values forest plot. (**B**) Sensitivity analysis of BMI Z-score forest plot (O’Connor et al. [32] study not included). CI, confidence interval.

Additionally, a second analysis including only children with obesity [31,32,36,38] showed that protocols did not present a significant effect on the intervention (MD −0.01; CI −0.04 to 0.02; *P* = 0.53; I^2^ = 80%) (Figure 3A). However, after the sensitivity analysis excluding the O′Connor study [32], a significant effect was found in BMI reduction (MD −0.09; CI −0.15 to −0.03; *P* = 0.002; I^2^ = 55%). Farpour-Lambert et al. [36] showed the highest proportion in this sub-analysis (92.1%), compared to 4% in the study by Wafa et al. [38] and 3.9% in the Davis et al. study [31] (Figure 3B).

#### 3.4.2. Engagement in Physical Activity

**Figure 4:**
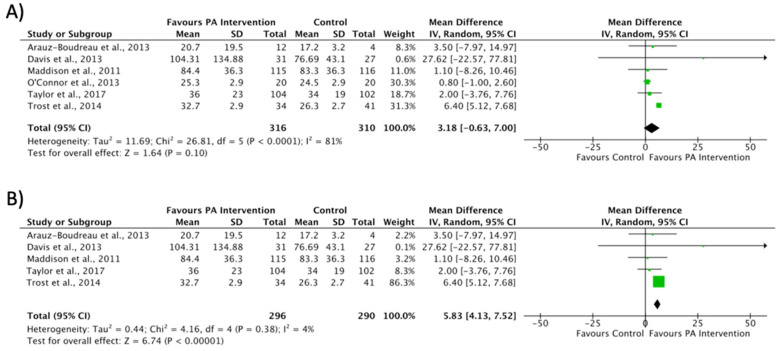
Effect interventions on increased moderate to vigorous physical activity time in minutes. (**A**) Moderate to vigorous physical activity levels forest plot. (**B**) Sensitivity analysis of moderate to vigorous physical activity forest plot (O’Connor et al. [32] not included). CI, confidence interval.

Regarding MVPA, the meta-analysis did not show effects on increasing the minutes of PA in the intervention groups compared with the control groups, although there was a high heterogeneity (MD 3.18 min; CI −0.63 to 7.00; *P* = 0.10; I² = 81%) (Figure 4A). After the sensitivity analysis excluded the O’Connor study [32], the meta-analyses showed a significant difference in the intervention and a reduction of heterogeneity without changing the direction of the outcome (MD 5.83 min; CI 4.13 to 7.52; *P* < 0.001; I² = 4%) (Figure 4B). Only one study showed significant changes following the intervention [33], with an increase in MVPA minutes in the intervention group with respect to the non-intervention group. In addition, Trost et al. [33] reported the greatest weight in the analysis (86.3%).

## 4. Discussion

The present meta-analysis provides evidence regarding the effectiveness of interventions with a PA component and lifestyle recommendations to reduce BMI Z-score in studies including children with obesity by increasing MVPA engagement. Increases were found in MVPA time in intervention groups compared with the control groups in the RCTs. However, these results should be interpreted with caution because the study by O′Connor et al. [32] must be excluded from the sensitivity analysis, due to the heterogeneity in the effect of the intervention, resulting in an outlier case of heterogeneity in the model. Nevertheless, forest plots shown in the meta-analysis before and after the sensitivity analysis indicate there was no variation in the direction of the meta-analysis effect in any of the cases. The only exception was in the PA intervention subgroups which included a nutritional recommendation; these changed the direction of the control group toward the intervention effect (Figure 2 and Figure 3).

The Tremblay et al. meta-analysis [52] reported significant reductions in BMI which were associated with a lower sedentary time. However, there are some limitations to this paper which must be considered. Firstly, children and adolescents (5 to 17 years old) were considered together, which may present a confounding factor in this analysis. Adolescents do not usually demonstrate the same habits or lifestyle as school children, with the latter experiencing greater parental control over their activities. Moreover, the intervention′s duration appears to be crucial when determining their effectiveness with regard to BMI changes. Our analysis was carried out on controlled and intervention groups based on mean differences among basal and post-intervention time using the variables BMI Z-score and MVPA time. Non-significant results, similar to those found by Nooijen et al. [7], were reached. Therefore, thereafter, the effect of PA interventions on how to reduce these variables was examined, selecting only post-intervention data for school-age children.

Considering the studies included in the present meta-analysis that reported validated data for this anthropometric variable, between 3 and 6 months are required to reduce BMI Z-score following involvement in a PA intervention [30,33,34,36,38]. These results are reinforced after the sub-analysis considering only studies with children with obesity [31,32,36] in which Farpour-Lambert et al. [36] represents 92.1% of the analysis weight. Therefore, it seems that engaging in three 60 min sessions [36] of controlled training by a physical education instructor a week is enough to begin a BMI Z-score improvement.

Combining an adequate time spent engaging in PA with an adequate intensity may lead to better changes—for example, a study combining aerobic and strength exercises showed a decrease in BMI [36]. In the present work, Trost et al. [33] showed the greatest weight in the analysis with the lowest BMI values after a PA intervention compared with the control group. This may be due combining PA practice (minimum of 60 min MVPA daily) with AVG. AVG have been suggested as a useful tool for increasing MVPA within the obese pediatric population and encouraging parents to substitute passive video games for these active games [34]. This tool seems to show a positive change from short to moderate PA time in participants aged between 3 and 17 years [53]. However, it is not clear if AVG are appropriate for reducing sedentary habits or whether they might promote increased passive gaming time. In addition, other protocols whose aims only included PA components [30] or telemedicine [31] did not find changes in BMI. Therefore, it seems that interventions not carried out under the direct supervision of a physical education instructor present lower effectiveness than online or indirect actions.

Nutritional intervention combined with PA intervention is another factor to be considered in the treatment of pediatric obesity. Four of the studies [32,35,37,38] included in the present meta-analysis reported good obesity control results despite the majority only including nutritional recommendations and not specific controlled interventions. The O’Connor’s study [32] was based on giving families nutritional advice but offered no limitations of specific foods. Wafa et al. [38] also gave parents behavioral change techniques during educational sessions, and Taylor et al. [35] proposed dietary objectives evaluated by questionnaires which obtained a specific healthy intake pattern score. Cliff et al. [37] included a dietary modification program, thus allowing parents to improve food behavior and diet quality.

A combination of PA and nutritional programs has been described as more effective at improving adiposity [54] or BMI Z-score [55], relative to PA or interventions with nutritional recommendations when delivered individually. A recent meta-analysis has also shown greater effectiveness through multidisciplinary interventions incorporating both nutrition and PA when delivered to overweight/obese children [13,56], although the weight of each intervention has not been shown. In our meta-analysis, the results obtained based on a BMI Z-score reduction showed a significant global effectiveness (Figure 2 and Figure 3). However, of the PA interventions with a complementary nutritional recommendation, only two studies [35,38] in the nutritional subgroup showed non-significant effectiveness. Therefore, PA seems to be an important component in BMI reduction in children with obesity.

In relation to the increase in MVPA practice, an intervention duration of between 4 and 6 months [30,33,34] generates an increase in minutes of engagement and intensity levels in children with obesity. Moreover, the effects of PA seem to be maintained long-term in interventions of up to 24 months [35]. Trost et al. [33] showed the greatest weight in the meta-analysis in favor of the intervention by increasing MVPA min in children with obesity, with an intervention based on an AVG program. It would be interesting to study how long the effects achieved by the AVG program last over a longer period of time. In addition, it would have been interesting to investigate whether these children adhere to outdoor PA, given that children’s motor skills and muscular strength development could be improved [57].

## 5. Limitations

There is very limited literature [31,32,36,38] focusing solely on obese school-aged children, regarding the number of PA component interventions and objectively accelerometry measured PA, which is recognized by the authors as a possible limitation. This meta-analysis incorporated studies where PA intervention was generally accompanied by other interventional components (Table 1), the reason being that the treatment of childhood obesity cannot be solely based on PA—it must be a multidisciplinary treatment including nutritional and motivational interventions. Moreover, many of the studies focused on lifestyle interventions, including different BMI groups or age groups. Therefore, the present meta-analysis is based principally on PA and BMI Z-score changes, although there are other (possible) susceptible factors to be included in the interventions. These studies may influence the participant´s level of BMI reduction acting as cofounding factors compared with PA only interventions; however, this subgroup analysis was shown in the forest plot.

Further, the mean reduction effect for the BMI Z-Score was (−0.09); although it produced a significant effect in meta-analysis results, this absolute value difference may not be clinically significant in treating childhood obesity.

## 6. Conclusions

In conclusion, this meta-analysis supports the notion that interventions with a PA component in school children with obesity seem to be successful in reducing BMI and produce an increase in time spent engaged in PA, especially MVPA. Therefore, interventions based on PA should be considered one of the main strategies to be implemented to fight childhood obesity.

## Figures and Tables

**Table 1:** Characteristics of selected studies by interventions content physical activity and accelerometry [30,31,32,33,34,35,36,37,38,39].

Study	Group	Sample (n)	Included Only Obese Children (Yes/No) /Obese Children (%)	Gender (%F)	Treatment Length	Intervention Type	Treatment Content PA	Accelerometer	Accelerometer Time	Author Accelerometer Protocol
Arauz-Boudreau et al. (2013)	Control Intervention	12 14	No/NR	61%	6 months	Lifestyle educational classes and PA training sessions	5 consecutive weekly sessions, increased to six 3 months later.	ActiGraph GT1M	Complete week; ≥8 hours on ≥4 days	Trost, 2005
Cliff et al. (2011)	Control Intervention	42 60	No/78%	58%	12 months	PA skill development program and dietary modification programs aimed at parents	6 months intervention divided into two components: 10-week face to face and 3 months with minimal contact. The first phase of treatment included weekly 2 h group sessions (90 min PA per session) and weekly ‘‘home challenge’’ activities.	ActiGraph 7164	≥8 consecutive days during waking hours	Freedson, 1997
Davis et al. (2013)	Control Intervention	27 31	Yes/100%	39%	8 months	Multidisciplinary actions based on psychoeducational group sessions delivered via telemedicine	Session over the phone with parents to encourage healthy habits. 1-h duration for 8 weeks	ActiGraph	Complete week; ≥6 h on ≥3 days	Sirard, 2000
Farpour-Lambert et al. (2009)	Control Intervention	22 22	Yes/100%	64%	3 months (3 months post-intervention)	PA training sessions	The exercise group trained 60 min 3 times/week for 3 months.	ActiGraph MT 6471	Complete week; 24 h/day on ≥4 days (including 1 weekend day)	Ekelund, 2001
Maddison et al. (2011)	Control Intervention	162 160	No/NR	37%	6 months	Active video games	Children were encouraged to meet the current PA recommendations by supplementing periods of inactivity or substituting periods of traditional non-active video games for playing AVG.	ActiGraph AM7164-2.2C	Complete week	Freedson, 2005
O’Connor et al. (2013)	Control Intervention	20 20	Yes/100%	80%	7 months	Healthy family activity and nutritional recommendations	Promote healthy child lifestyle behavior with effective behavior-specific parenting practices	ActiGraph 7064	≥5 days; ≥800 min/day	Treuth, 2004
Serra-Paya et al. (2015)	Control Intervention	59 54	No/NR	44%	8 months	PA sessions, theoretical and practical sessions for parents, behavior strategy sessions for both children and parents, and weekend activities.	90 min PA session—3 ses/w for 8 months	ActiGraph GT3X+	≥8 consecutive days during waking hours	NR
Taylor et al. (2015)	Control Intervention	102 104	No/NR	55%	24 months	Family-based intervention with specific goals based on dietary intake and PA	Families attended monthly multidisciplinary sessions to develop specific goals suitable for the first year. Thereafter, every third month for another 12 months to discuss progress and provide support.	ActiGraph GT3X	Complete week; ≥8 hours on ≥4 days	Evenson, 2008
Trost et al. (2014)	Control Intervention	41 34	No/35,7%	55%	4 months	Lifestyle educational parents and programmed active gaming	24 session topics about aspects of nutrition, sleep hygiene, PA, screen time, and AVG 60 min PA AVG session	ActiGraph GT3X / GT3X+	Complete week; ≥9 h on ≥3 days	Evenson, 2008
Wafa et al. (2011)	Control Intervention	55 52	Yes/100%	50%	6 months	Information based nutrition and health education	60 min PA intervention sessions (8 sessions for 6 months)	ActiGraph GT1M	5 days; ≥10 h on ≥4 days	Reilly, 2003
										Puyau, 2002

AVG, active video games; F, female; NR, not referred; PA, physical activity; ses/w, sessions per week.

## Supplementary Materials

The following are available online at https://www.mdpi.com/1660-4601/17/17/6031/s1, Figure S1: Assessment of risk of bias, Table S1: PRISMA Checklist, Appendix: Research strategy.
