# Supplementary material for: Evaluation of Physical Activity and Lifestyle Interventions Focused on School Children with Obesity Using Accelerometry: A Systematic Review and Meta-Analysis
Source: Int J Environ Res Public Health. 2020 Aug 19;17(17):6031. doi: 10.3390/ijerph17176031 (PMC7503305; doi:10.3390/ijerph17176031)
Supplement: Supplementary file 1 [file ijerph-17-06031-s001.pdf]

Supplementary Materials

Evaluation of physical activity and lifestyle interventions focused on school children with obesity using accelerometry: a systematic review and meta-analysis

Jose Manuel Jurado-Castro[1], Mercedes Gil-Campos[1,2,3], Hugo Gonzalez-Gonzalez[4], Francisco Jesus Llorente-Cantarero[1,2,5]

Contents

Table S1. PRISMA Checklist
Figure S1. Assessment of risk of bias
Appendix. Research strategy

Table S1. PRISMA Checklist

Section/topic	#	Checklist item	Reported on page #
**TITLE**			
Title	1	Identify the report as a systematic review, meta-analysis, or both.	#1
**ABSTRACT**			
Structured summary	2	Provide a structured summary including, as applicable: background; objectives; data sources; study eligibility criteria, participants, and interventions; study appraisal and synthesis methods; results; limitations; conclusions and implications of key findings; systematic review registration number.	#1
**INTRODUCTION**			
Rationale	3	Describe the rationale for the review in the context of what is already known.	#2
Objectives	4	Provide an explicit statement of questions being addressed with reference to participants, interventions, comparisons, outcomes, and study design (PICOS).	#2
**METHODS**			
Protocol and registration	5	Indicate if a review protocol exists, if and where it can be accessed (e.g., Web address), and, if available, provide registration information including registration number.	#2
Eligibility criteria	6	Specify study characteristics (e.g., PICOS, length of follow-up) and report characteristics (e.g., years considered, language, publication status) used as criteria for eligibility, giving rationale.	#3
Information sources	7	Describe all information sources (e.g., databases with dates of coverage, contact with study authors to identify additional studies) in the search and date last searched.	#3
Search	8	Present full electronic search strategy for at least one database, including any limits used, such that it could be repeated.	#3
Study selection	9	State the process for selecting studies (i.e., screening, eligibility, included in systematic review, and, if applicable, included in the meta-analysis).	#3
Data collection process	10	Describe method of data extraction from reports (e.g., piloted forms, independently, in duplicate) and any processes for obtaining and confirming data from investigators.	#3
Data items	11	List and define all variables for which data were sought (e.g., PICOS, funding sources) and any assumptions and simplifications made.	#3
Risk of bias in individual studies	12	Describe methods used for assessing risk of bias of individual studies (including specification of whether this was done at the study or outcome level), and how this information is to be used in any data synthesis.	#3
Summary measures	13	State the principal summary measures (e.g., risk ratio, difference in means).	#4

Section/topic	#	Checklist item	Reported on page #
Synthesis of results	14	Describe the methods of handling data and combining results of studies, if done, including measures of consistency (e.g., I^2) for each meta-analysis.	#4

Section/topic	#	Checklist item	Reported on page #
Risk of bias across studies	15	Specify any assessment of risk of bias that may affect the cumulative evidence (e.g., publication bias, selective reporting within studies).	#4
Additional analyses	16	Describe methods of additional analyses (e.g., sensitivity or subgroup analyses, meta-regression), if done, indicating which were pre-specified.	#4
**RESULTS**			
Study selection	17	Give numbers of studies screened, assessed for eligibility, and included in the review, with reasons for exclusions at each stage, ideally with a flow diagram.	#4
Study characteristics	18	For each study, present characteristics for which data were extracted (e.g., study size, PICOS, follow-up period) and provide the citations.	#5,6,7,8
Risk of bias within studies	19	Present data on risk of bias of each study and, if available, any outcome level assessment (see item 12).	#9
Results of individual studies	20	For all outcomes considered (benefits or harms), present, for each study: (a) simple summary data for each intervention group (b) effect estimates and confidence intervals, ideally with a forest plot.	#9,10,11
Synthesis of results	21	Present results of each meta-analysis done, including confidence intervals and measures of consistency.	#9,10,11
Risk of bias across studies	22	Present results of any assessment of risk of bias across studies (see Item 15).	#9
Additional analysis	23	Give results of additional analyses, if done (e.g., sensitivity or subgroup analyses, meta-regression [see Item 16]).	#9,10,11
**DISCUSSION**			
Summary of evidence	24	Summarize the main findings including the strength of evidence for each main outcome; consider their relevance to key groups (e.g., healthcare providers, users, and policy makers).	#11,12,13
Limitations	25	Discuss limitations at study and outcome level (e.g., risk of bias), and at review-level (e.g., incomplete retrieval of identified research, reporting bias).	#12,13
Conclusions	26	Provide a general interpretation of the results in the context of other evidence, and implications for future research.	#13
**FUNDING**			
Funding	27	Describe sources of funding for the systematic review and other support (e.g., supply of data); role of funders for the systematic review.	n/a

From: Moher D, Liberati A, Tetzlaff J, Altman DG, The PRISMA Group (2009). Preferred Reporting Items for Systematic Reviews and Meta-Analyses: The PRISMA Statement. PLoS Med 6(7): e1000097. doi:10.1371/journal.pmed1000097

Figure S1. Assessment of risk of bias

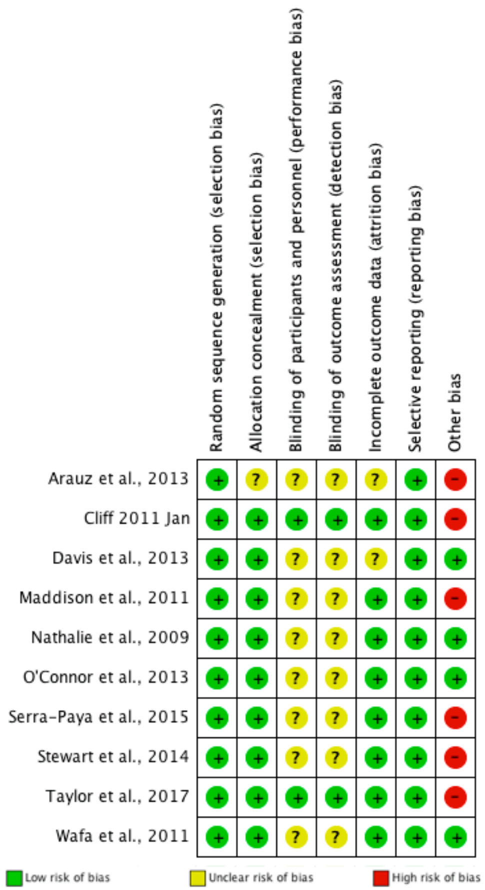

Appendix

1. MEDLINE (PubMed) Field Tags: title & abstract

Date of Search: 2018-08-31
Publication years: 1991 to date
Number of Hits: 222

((((("exercise"[MeSH Terms] OR "exercise"[All Fields]) OR ("physical"[All Fields] AND "activity"[All Fields])) OR "physical activity"[All Fields]) AND ("accelerometry"[MeSH Terms] OR "accelerometry"[All Fields])) AND (("pediatric obesity"[MeSH Terms] OR ("pediatric"[All Fields] AND "obesity"[All Fields])) OR "pediatric obesity"[All Fields])) AND (((((("child"[MeSH Terms] OR "child"[All Fields]) OR "children"[All Fields]) OR "child s"[All Fields]) OR "children s"[All Fields]) OR "childrens"[All Fields]) OR "childs"[All Fields])

Translations

physical activity: "exercise"[MeSH Terms] OR "exercise"[All Fields] OR ("physical"[All Fields] AND "activity"[All Fields]) OR "physical activity"[All Fields]

accelerometry: "accelerometry"[MeSH Terms] OR "accelerometry"[All Fields]

pediatric obesity: "pediatric obesity"[MeSH Terms] OR ("pediatric"[All Fields] AND "obesity"[All Fields]) OR "pediatric obesity"[All Fields]

child: "child"[MeSH Terms] OR "child"[All Fields] OR "children"[All Fields] OR "child's"[All Fields] OR "children's"[All Fields] OR "childrens"[All Fields] OR "childs"[All Fields]

2. Cochrane Register of Controlled Trials (CENTRAL) Field Tags: All text

Date of Search: 2018-08-31
Publication years: 1991 to date
Number of Hits: 89

---

1. physical activity
   AND
2. accelerometry
   AND
3. child
   AND
4. pediatric obesity

3. Web of Science Field Tags: TOPIC

Date of Search: 2018-08-31
Publication years: 1991 to date
Number of Hits: 1411

TOPIC:(physical activity) AND TOPIC:(acceleromet*) AND TOPIC: (child*) AND TOPIC: (obes*)
Refined by: DOCUMENT TYPES: (ARTICLE)
Indexes: SCI-EXPANDED, SSCI, A&HCI, CPCI-S, CPCI-SSH, BKCI-S, BKCI-SSH, ESCI, CCR-EXPANDED, IC.

Complemmentary Search

4. ScienceDirect (SCOPUS) Field Tags:

Date of Search: 2018-08-31
Publication years: 1991 to date
Number of Hits: 207

Physical activity AND accelerometer AND child AND pediatric obesity
Filtered by: research articles

5. PROQuest Field Tags:

Date of Search: 2018-08-31
Publication years: 1991 to date
Number of Hits: 2600

Physical activity AND acceleromet* AND child* AND pediatric obesity
Filtered by: research articles

6. BVS (Biblioteca Virtual en Salud)	Field Tags: título, resumen, asunto
Date of Search: 2018-08-31	
Publication years: 1991 to date	
Number of Hits: 115	

tw:((tw:(physical activity)) AND (tw:(accelerometry)) AND (tw:(child)) AND (tw:(pediatric obesity))) AND (year_cluster:[1991 TO 2018]

7. Annual Reviews Field Tags:

Date of Search: 2018-08-31
Publication years: 1991 to date
Number of Hits: 2

Physical activity AND accelerometer AND child AND pediatric obesity

8. LILACS (Literatura Latino americana y del Caribe en CC de la Salud)	Field Tags:

Date of Search: 2018-08-31
Publication years: 1991 to date
Number of Hits: 3

physical activity AND accelerometry AND child AND pediatric obesity AND (db:("LILACS"))

9. Dialnet Field Tags:

Date of Search: 2018-08-31
Publication years: 1991 to date
Number of Hits: 0

Actividad física AND acelerometria AND niños AND obesidad

10. Scielo. Field Tags: All indexes

Date of Search: 2018-08-31
Publication years: 1991 to date
Number of Hits: 7

---

1. physical activity AND accelerometer AND child AND pediatric obesity
2. actividad física AND acelerometria AND niños AND obesidad
